# Herbal formula LLKL ameliorates hyperglycaemia, modulates the gut microbiota and regulates the gut‐liver axis in Zucker diabetic fatty rats

**DOI:** 10.1111/jcmm.16084

**Published:** 2020-11-20

**Authors:** Mei Li, Lei Ding, Yu‐Li Hu, Ling‐Ling Qin, You Wu, Wei Liu, Li‐Li Wu, Tong‐Hua Liu

**Affiliations:** ^1^ Key Laboratory of Health Cultivation of the Ministry of Education Dongfang Hospital Beijing University of Chinese Medicine Beijing China; ^2^ Key Laboratory of Health Cultivation of the Ministry of Education School of Traditional Chinese Medicine Beijing University of Chinese Medicine Beijing China; ^3^ Tibetan Medical College Lhasa China

**Keywords:** gut microbiota, gut‐liver axis, lipopolysaccharide, T2DM, toll‐like receptor signalling pathway, traditional Chinese medicine, transcriptome

## Abstract

LLKL, a new traditional Chinese medicine formula containing *Edgeworthia gardneri* (Wall.) Meisn., *Sibiraea angustata* and *Crocus sativus* L. (saffron), was designed to ameliorate type 2 diabetes mellitus. Despite the therapeutic benefits of LLKL, its underlying mechanisms remain elusive. This study evaluated the LLKL anti‐diabetic efficacy and its effect on gut microbiota to elucidate its mechanism of action in Zucker diabetic fatty rats. We found that administration of different LLKL concentrations (4.68, 2.34 and 1.17 g/kg/d) improved several diabetic parameters after a 6‐week treatment. Moreover, LLKL modulated gut microbiota dysbiosis, increased the expression of occluding and maintained intestinal epithelial homeostasis, leading to a reduction in LPS, TNF‐α and IL‐6 levels. Hepatic transcriptomic analysis showed that the Toll‐like receptor signalling pathway was markedly enriched by LLKL treatment. RT‐qPCR results validated that LLKL treatment decreased the expressions of TLR4, MyD88 and CTSK. Furthermore, a gene set enrichment analysis indicated that LLKL enhanced the insulin signalling pathway and inhibited glycerolipid metabolism and fatty acid metabolism, which were verified by the liver biochemical analysis. These findings demonstrate that LLKL ameliorates hyperglycaemia, modulates the gut microbiota and regulates the gut‐liver axis, which might contribute to its anti‐diabetic effect.

## 1 INTRODUCTION

Type 2 diabetes mellitus (T2DM), a chronic metabolic disorder due to insulin resistance and insufficient insulin secretion,^1^ is becoming a major global public health issue.^2^ Over the past decades, the number of adults with diabetes has increased from 108 million in 1980 to 463 million in 2019 worldwide.^3^, ^4^ According to the data from the International Diabetes Federation, the incidence of diabetes mellitus continues to increase and will reach 700 million individuals by 2045,^4^ of which T2DM accounts for more than 90%.^4^ T2DM is characterized by hyperglycaemia,^5^ hyperinsulinaemia^6^ and impaired glucose and lipid metabolisms in the liver,^5^ accompanied by chronic low‐grade inflammation.^7^

Accumulating evidence indicates that an altered gut microbiota composition and diversity contribute to the onset and progression of diabetes.^8^ For example, a higher *Firmicutes*/*Bacteroidetes* ratio is always observed in both diabetic patients and animals, which further leads some substances, such as lipopolysaccharide (LPS), to impair the gut barrier function.^9^, ^10^, ^11^ A lower expression of the gut barrier protein occludin was also observed in diabetic mice, indicating an increase in intestinal permeability.^12^ LPS penetrates the intestinal epithelium into the blood circulation and liver via the portal circulation, leading to the activation of the mononuclear macrophage system and further regulates release of various cell “toxic factors”, such as cytokines, inflammatory mediators, proteases and oxygen‐free radicals.^13^, ^14^

The gut‐liver axis communicates with organs of the digestive system through the biliary tract, portal vein and systemic crosstalk, which promotes the gut factors to regulate liver glucose and lipid metabolisms.^15^ One major pathway by which the gut microbiota regulates the glycaemic control, inflammatory response and liver metabolism is the Toll‐like signalling pathway.^16^ In the liver, LPS binds to Toll‐like receptors located on the membrane of hepatic cells and activates key signalling pathways, such as the myeloid differentiation primary response gene 88 (MyD88),^17^ directly inducing the transcription of pro‐inflammatory cytokines, including interleukin‐6 (IL‐6) and tumour necrosis factor‐α (TNF‐α), which then favours the insulin resistance.^18^ Furthermore, a recent study also reported that cathepsin K (CTSK) could be up‐regulated by an imbalance of microbiota and might function through Toll‐like receptor 4 (TLR4) to up‐regulate inflammatory factors.^19^ The metabolic inflammation mediated insulin resistance through the inhibition of insulin signalling, which suppressed the hepatic glucose production or induced the production of ‘‘second messengers’’, such as fatty acids. It also stimulated hepatic lipogenesis, contributing to steatosis and elevated serum lipid levels.^20^ Thus, the modulation of gut microbiota is a promising and feasible target for treating T2DM.

Traditional Chinese medicine (TCM) has been applied to the treatment of metabolic diseases such as diabetes and obesity for many years.^18^ LLKL, a new TCM formula (patent number: 201610922905.3) containing *Edgeworthia gardneri* (Wall.) Meisn., *Sibiraea angustata* and *Crocus sativus* L. (saffron), was designed to ameliorate T2DM. The flower of *Edgeworthia gardneri* (Wall.) Meisn., also called “lvluohua” in Tibet, China, has been widely used as a folk medicine to prevent and treat inflammation, cardiovascular disease and various metabolic diseases, including diabetes and hyperlipidaemia.^21^ *Sibiraea angustata*, known as “liucha” and used as a common and civil traditional medicine in Tibet, China, contains many active components, such as terpenes, phenolic acid, saponins and polysaccharides,^22^ with significant lipid‐lowering and anti‐obesity activity.^23^, ^24^ Moreover, saffron consumption combined with exercise reportedly improved diabetic parameters through redox‐mediated mechanisms and stimulated the GLUT4/AMPK pathway to enhance glucose uptake.^25^ In light of the above data, we hypothesized that the LLKL formula has more promising anti‐diabetic benefits.

The present study evaluated the anti‐diabetic therapeutic effectiveness and the underlying mechanisms of LLKL in Zucker diabetic fatty (ZDF) rats. We explored LLKL influence on insulin resistance and inflammatory status and assessed its capability to modulate the gut microbiota, hepatic glucose and lipid metabolisms, and the gut‐liver axis activation via the Toll‐like receptor signalling pathway. Our findings present LLKL as a promising drug for the treatment of T2DM.

## 2 MATERIALS AND METHODS

### 2.1 Preparation of LLKL mixture

LLKL was prepared with three herbs, *Edgeworthia gardneri* (Wall.) Meisn., *Sibiraea angustata* and *Crocus sativus* L. (saffron) at a ratio of 15:10:1 (w/w/w). *Edgeworthia gardneri* (Wall.) Meisn. was purchased from a local medical farmer's market in Yunnan, China. *Sibiraea angustata* was collected in Tibet and identified by Professor Lan Cao, Jiangxi University of Traditional Chinese Medicine in China. *Crocus sativus* L. was obtained from Esfedan Saffron Co. (Mashhad, Iran). To prepare this formula, the flowers of *Edgeworthia gardneri* (Wall.) Meisn. and *Sibiraea angustata* were soaked in a 10‐time volume of distilled deionized water, respectively, for 30 minutes and decocted twice for 60 and 45 minutes after boiling. *Crocus sativus* L. was reflux‐extracted with a 10‐time volume of 70% ethanol twice for 60 and 45 minutes after boiling. After filtration and collection, the supernatants of *Edgeworthia gardneri* (Wall.) Meisn., *Sibiraea angustata* and *Crocus sativus* L. (saffron) were concentrated under reduced pressure and then lyophilized by a vacuum concentration, respectively. Finally, for the animal experiment, the extracts of *Edgeworthia gardneri* (Wall.) Meisn., S*ibiraea angustata* and *Crocus sativus* L. (saffron), were mixed and dissolved in distilled deionized water. The final doses used in our study were LLKL_H (4.68 g/kg/d), LLKL_M (2.34 g/kg/d), LLKL_L (1.17 g/kg/d), *Edgeworthia gardneri* (Wall.) Meisn. (1.35 g/kg/d), *Sibiraea angustata* (0.9 g/kg/d) and *Crocus sativus* L. (saffron) (0.09 g/kg/d). The dose of LLKL_M group was equivalent to approximately a dose of 26 g/d/person in humans, according to an extrapolation performed using the body surface area normalization method. The doses of *Edgeworthia gardneri* (Wall.) Meisn., *Sibiraea angustata* and *Crocus sativus* L. (saffron) groups were the same as those in the LLKL_M group.

### 2.2 Analysis of LLKL chemical profile

Chromatographic separation of LLKL constituents was performed using an ACQUITY UPLC BEH C18 column (2.1 × 50 mm, 1.7 μm) at 40°C. Mobile phases A and B comprised acetonitrile and 0.2% formic acid in water, respectively. The elution condition was applied with a gradient programme as follows: 0 minute, 10% A; 0.3 minute, 10% A; 4 minutes, 99% A; 4.3 minutes, 99% A; 4.4 minutes, 10% A; 5.0 minutes 10% A. ESI source conditions were set as follows: capillary voltage of 3.20 kV (positive), cone voltage of 20 V, source temperature of 110°C, desolvation temperature of 400°C, cone gas flow of 50 L/h, desolvation gas flow of 900 L/h, collision gas flow of 0.28 mL/min and collision energy of 20/30/40 V. 3 μL of the LLKL sample was injected into the Hclass‐vion IMS QTof (Waters) system for analysis.

### 2.3 Animals and experimental design

**Figure 1:**
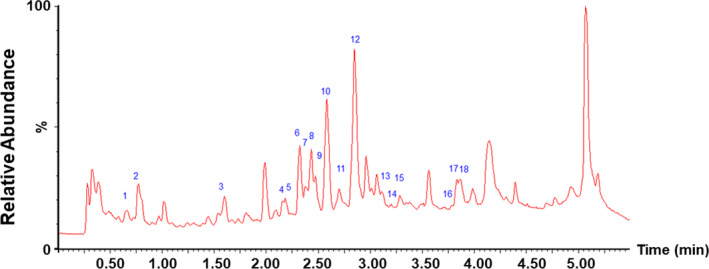
Base peak chromatogram of LLKL in positive mode. Each peak number is consistent with Table 2

**Figure 2:**
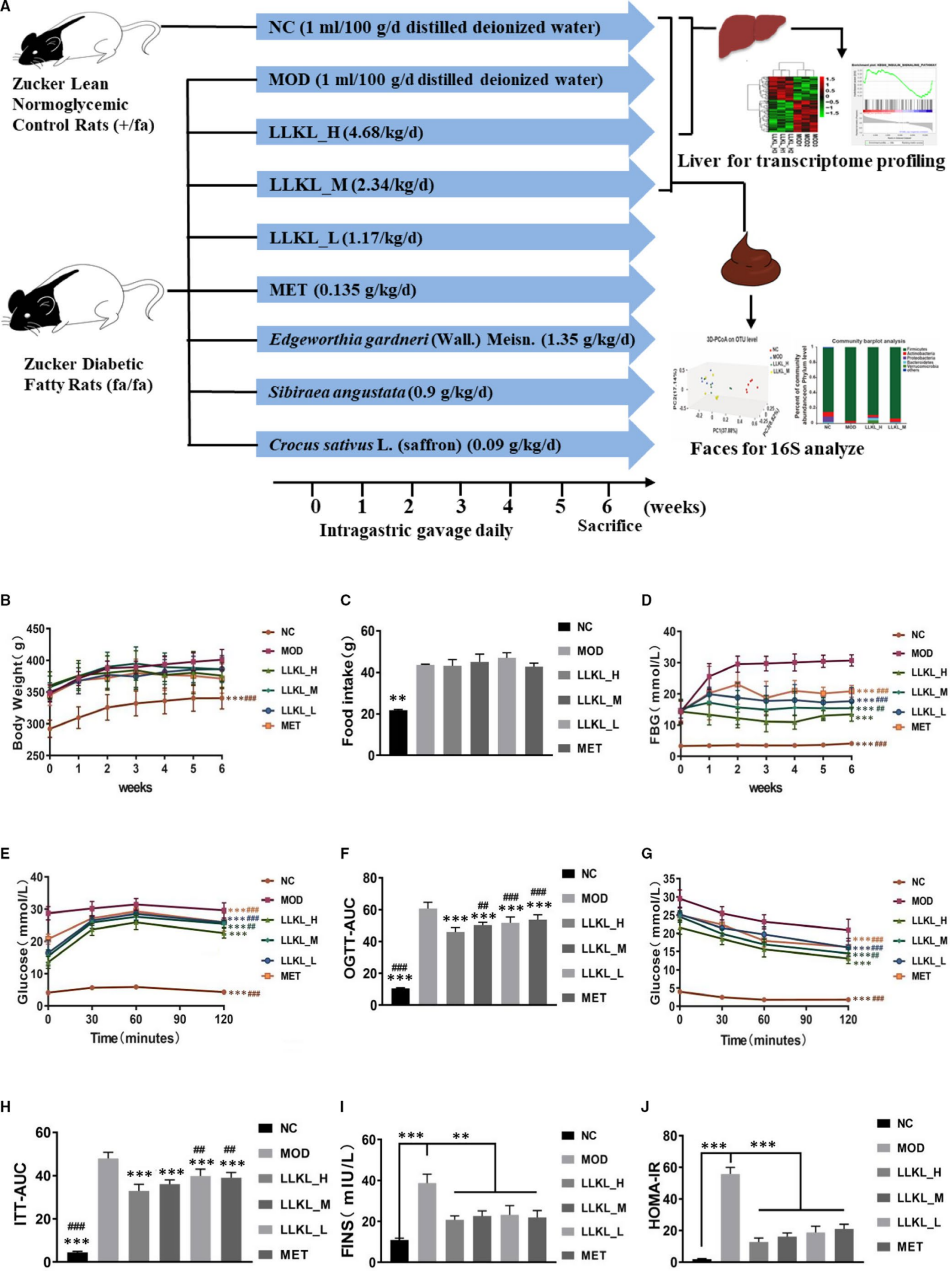
LLKL treatment alleviates insulin resistance in ZDF rats. A, Schematic diagram of the animal experiment. B, BW, (C) food intake, (D) FBG, (E) curve of OGTT, (F) AUC of OGTT, (G) curve of ITT, (H) AUC of ITT, (I) FINS and (J) HOMA‐IR of NC, MOD, LLKL_H, LLKL_M, LLKL_L and MET treatment groups. Data were shown as means ± SD (n = 8, *P < .05, **P < .01, ***P < .001 vs the MOD group; ^#^ *P* < .05, ^##^ *P* < .01, ^###^ *P* < .001 vs the LLKL_H group)

Male Zucker lean normoglycaemic rats (ZLN, +/fa, 13‐14 weeks) and male Zucker diabetic fatty rats (ZDF, fa/fa, 13‐14 week), which have already been administrated of purina#5008 diet for 4 weeks in order to induce T2DM status, were obtained from Beijing Vital River Laboratory Animal Technology Co., Ltd (licence number: SYXK (Jing) 2016 0011). Rats were housed at a temperature of 25 ± 2°C, relative humidity of 50 ± 5% and a 12/12‐hour day‐night cycle under specific pathogen‐free conditions. All rats had free access to food and water. After 2 weeks of adaptive breeding, the fasting blood glucose (FBG) and bodyweight (BW) of each rat were measured. ZDF rats with FBG ≥ 7.8 mmol/L^26^ were considered diabetic and used in the subsequent experiments. According to the FBG and BW values, diabetic rats were then randomly assigned to each of the following 8 groups: diabetic model group (MOD, n = 8), LLKL high‐dose group (LLKL_H, n = 8), LLKL middle‐dose group (LLKL_M, n = 8), LLKL low‐dose group (LLKL_L, n = 8), *Edgeworthia gardneri* (Wall.) Meisn. group (n = 8), *Sibiraea angustata* group (n = 8), *Crocus sativus* L. (saffron) group (n = 8) and metformin group (MET, n = 8). ZLN rats were used as the normal control group (NC, n = 8). Metformin (Sino‐American Shanghai Squibb Pharmaceutical Ltd, Shanghai China) at a dose of 0.135 g/kg/d was used as the positive control to evaluate the hypoglycaemic effectiveness in this study. BW and FBG were measured weekly. After 6 weeks, oral glucose tolerance test (OGTT) and insulin tolerance test (ITT) were performed. Then, all rats were anaesthetized with 1% sodium pentobarbital (45 mg/kg), and the blood samples were collected and centrifuged at 3000 *g*, 4°C for 15 minutes to separate the serum. Faeces and liver and intestine tissues were quickly harvested and frozen in liquid nitrogen or fixed with 4% paraformaldehyde. This study was conducted in strict accordance with the Guide for Care and Use of Laboratory Animals of Beijing University of Chinese Medicine (Beijing, China). The schematic diagram of animal experiment is shown in Figure 1, 2.

### 2.4 Measurement of FBG, OGTT and ITT levels

Blood glucose level was measured in the tail vein blood using a blood glucose meter (ARKRAY). FBG was tested after deprivation of food for 12 hours overnight. OGTT and ITT were performed after a 6‐week treatment. Rats were fasted for 12 hours overnight and 4 hours before performing the OGTT and ITT, respectively. We detected blood glucose levels at 0, 30, 60 and 120 minutes after administering 50% oral glucose (2 g/kg, BW) or subcutaneous injection of insulin (0.75 UI/kg, BW). The area under the curve (AUC) was calculated using the formula: AUC = 0.5 × (Bg 0 minute + Bg 30 minutes)/2 + 0.5 × (Bg 30 minutes + Bg 60 minutes)/2 + 1 × (Bg 60 minutes + Bg 120 minutes)/2, Bg indicated the blood glucose level at each time point.

### 2.5 Measurement of serum fasting insulin (FINS), FFA, LPS, TNF‐α and IL‐6 levels

Serum level of FFA was measured by automatic biochemical analyser (Beckman) using standard laboratory methods. FFA reagent was obtained from BioMérieux (Beckman). Serum levels of FINS (Abcam), LPS (Cusabio), TNF‐α (Abcam) and IL‐6 (Abcam) were measured using ELISA kits according to the manufacturer's instructions. The levels of TC and TG in the liver tissues were measured using TC and TG kits purchased from Nanjing Jiancheng Bioengineering Institute (Nanjing, China), following the manufacturer's instructions.

### 2.6 Haematoxylin and eosin (H&E) and periodic acid‐schiff (PAS) stainings

Fresh liver tissues and small intestine tissues were harvested from the rats, fixed in 4% paraformaldehyde (Solarbio) and embedded in paraffin. Prepared liver and small intestine slides were stained with haematoxylin and eosin (H&E) staining. Glycogen was measured using a PAS kit (Solarbio) following the manufacturer's instructions.

### 2.7 Oil Red O staining

For Oil Red O staining, fresh liver tissues were dissected and embedded in OCT. The Oil Red O staining was performed using the Oil Red O staining solution (Solarbio) following the manufacturer's instructions.

### 2.8 Immunohistochemical (IHC) staining and analysis

For IHC staining, small intestine paraffin tissues were sectioned into 5‐μm slices. Xylene was used for dewaxing and gradient ethanol was used for rehydration, respectively. The primary antibody of Occludin (ab216327, Abcam) was used. As described before,^27^ IHC staining was performed and the intensity of staining and the proportion of positive cells were used to evaluate the immunostaining.

### 2.9 Real‐time qPCR

After the total RNA was extracted and its concentration was measured, cDNA was synthesized using the HiScript II Q RT SuperMix for qPCR (+gDNA wiper) (Vazyme). Real‐time PCR was carried out by using the ChamQ SYBR qPCR Master Mix (Vazyme) and a LightCycler^®^ 480 II Real‐time PCR Instrument (Roche). The relative mRNA levels were normalized to ACTB. The mRNA relative quantitation was calculated using the ∆∆Ct method. The primers used are listed in Table 1.

**Table 1:** Primers used in RT‐qPCR

Genes	Forward primer 5′>3′	Reverse primer 5′>3′
*TLR4*	GAATGAGGACTGGGTGAGAAAC	ACCAACGGCTCTGGATAAAGT
*MyD88*	ATACGCAACCCAGCAGAAACAG	TATCATTGGGGCAGTAGCAGA
*FOS*	CGGTCAAGAAGATTAGCAACA	AGAAGGAACCTGACAGGTCCAC
*CTSK*	ACTCTGAAGACGCTTACCCG	CCTTTGCCGTGGCGTTATAC
*ACTB*	GGCACCACACTTTCTACAAT	GTCACACTTCATGATGGAGTTGAAGG

### 2.10 Gut microbiota analysis

The fresh faecal samples were collected from the NC, MOD, LLKL_M and LLKL_H groups after the 6‐week treatment and subsequently sent to Majorbio Biotech Co., Ltd. for 16S rDNA sequencing. The primers 338F (5′‐ACTCCTACGGGAGGCAGCA‐3′) and 806R (5′‐GGACTACHVGGGTWTCTAAT‐3′) were used to amplify the V3‐V4 variable regions of the 16S rDNA gene on a GeneAmp 9700 thermal cycler PCR system (Applied Biosystems). After the library was qualified, Illumina MiSeq PE300 platform was used for 16S rDNA sequencing. The data were analysed on the Majorbio Cloud Platform (www.majorbio.com). The original 16S rDNA sequencing raw data were deposited into the NCBI database (accession number: PRJNA604387).

### 2.11 Library preparation and illumina transcriptome sequencing

After extracting the total RNA from the liver tissues of the NC, MOD and LLKL_H groups, the RNA purity was determined using a NanoDrop 2000 spectrophotometer. The transcriptome strand library was prepared using the TruSeq™ stranded total RNA Kit from Illumina. After quantified by TBS380, the paired‐end RNA‐seq sequencing library was sequenced with the Illumina HiSeq xten (2× 150 bp read length) by Shanghai Majorbio Bio‐Pharm Biotechnology Co., Ltd. The raw data were deposited into the NCBI database (accession number: PRJNA601882).

### 2.12 Correlational and functional annotation analysis

After quality control, R version 3.4.1 cor was used to analyse the correlation between each two samples. Principal components analysis (PCA) of gene expression was performed with the R3.4.1 psych version 1.7.8. We then identified the differentially expressed genes (DEGs) with FDR < 0.05 and |logFC| > 0.5 by using the R3.4.1 limma (version 3.32.5).^28^ The DEGs heatmap was carried out using R3.4.1 pheatmap (version 1.0.8).^29^ DEGs were subjected to the DAVID 6.8 online tool^30^, ^31^ for gene ontology (GO) and Kyoto Encyclopedia of Genes and Genomes (KEGG) pathway analysis and the significant enrichment of DEGs was determined by a *P*‐value < .05, which was a modified Fisher exact *P*‐value.

### 2.13 Gene set enrichment analysis (GSEA) and gene‐pathway regulatory network analysis

GSEA is a computational method that determines whether a priori defined set of genes is statistically significant between two phenotypes.^32^ We adopted one kind of reported methods to perform GSEA.^33^, ^34^ Briefly, GSEA software version 4.02 was used. We employed the liver transcriptome expression profile data of NC, MOD and LLKL_H groups and the “phenotype label” includes MyD88 and CTSK, in which expression was verified to be down‐regulated by the LLKL_H treatment. GSEA generated an ordered list of all genes based on their association with MyD88 and CTSK expression, respectively. The Pearson correlation metric |*r*| > .25 was selected for the ranking genes. 1000 gene permutations were used to generate a null distribution for enrichment score, and then, each pathway will attain a normalization enrichment score (NES). The KEGG gene sets were used as the gene sets database. Gene sets enriched with |NES| > 1 were considered significant. The gene‐pathway regulatory network was subsequently constructed by connecting the “phenotype label gene”, “enriched pathway” and the related “co‐expression genes” based on the results of GSEA analysis using the cytoscape software.

### 2.14 Statistical analysis

Data were expressed as means ± SD with GraphPad Prism software (version 8.0). Two‐factor repeated‐measures ANOVA was performed for the FBG, BW, OGTT curve and ITT curve data analysis. Other data were analysed by 1‐way ANOVA. Statistical Package for Social Sciences (SPSS) software (version 23.0) was used for statistical analysis in this study. *P* < .05 was considered statistically significant.

## 3 RESULTS

### 3.1 Identification of chemical composition of LLKL

Hclass‐vion IMS QTof was used to analyse and identify the constituents of LLKL. As shown in Figure 2, we identified 18 phytochemicals, including 15 flavonoids and their glycosides and 3 organic acids. Table 2 shows the retention time, experimental mass and the identified compounds.

**Table 2:** The retention time, experimental mass and identified compounds

Peak	Component	Formula	RT (time)	Response
1	Luteolin‐7‐O‐β‐glucopyranoside	C_21_H_20_O_11_	0.658	63 116
2	Myristic acid	C_14_H_28_O_2_	0.692	2312
3	Baicalin	C_21_H_18_O_11_	1.591	47 286
4	Kaempferol	C_15_H_10_O_6_	2.190	226 507
5	Rutin	C_27_H_30_O_16_	2.191	171 233
6	Kaempferol‐3‐O‐rutinoside	C_27_H_30_O_15_	2.285	10 367
7	6‐Hydroxykaempferol	C_15_H_10_O_7_	2.316	133 778
8	6‐Hydroxykaempferol‐7‐O‐glucoside	C_21_H_20_O_12_	2.317	132 361
9	Safflor yellow A	C_27_H_30_O_15_	2.329	97 544
10	Safflomin C	C_30_H_30_O_14_	2.586	12 604
11	Quercetin‐3‐O‐α‐L‐rhamnoside	C_21_H_20_O_11_	2.610	21 433
12	Crocetin	C_20_H_24_O_4_	2.826	107 943
13	Dodecanoic acid	C_12_H_24_O_2_	3.171	11 290
14	Quercetin‐6‐O‐glucoside	C_21_H_20_O_13_	3.244	16 110
15	Quercetin	C_15_H_10_O_7_	3.260	6045
16	Apigenol	C_15_H_10_O_5_	3.764	2485
17	Hydroxysafflor yellow A	C_27_H_32_O_16_	3.956	12 028
18	Hexadecanoic acid	C_16_H_32_O_2_	3.994	2380

### 3.2 LLKL administration improves the glycaemic control and insulin resistance in ZDF Rats

To investigate the anti‐diabetic effects of LLKL and whether LLKL had a more beneficial effect than the individual herbs in LLKL, LLKL_H (4.68 g/kg/d), LLKL_M (2.34 g/kg/d), LLKL_L (1.17 g/kg/d), *Edgeworthia gardneri* (Wall.) Meisn. (1.35 g/kg/d), *Sibiraea angustata* (0.9 g/kg/d) and *Crocus sativus* L. (saffron) (0.09 g/kg/d) were orally administrated to ZDF rats for 6 weeks. MET and MOD were used as a positive control and the diabetic group, respectively. As shown in Figure 2B and Figure S1

A, BW of the MOD group was increased compared with the NC group, but BW of rats between any of the treatment groups and MOD group were not significantly different. In addition, no significant differences in the food intake between the MOD group and any of the treatment groups at the end of week 6 were observed (Figure 2C and Figure S1B). After LLKL_H, LLKL_M, LLKL_L, MET, *Edgeworthia gardneri* (Wall.) Meisn., *Sibiraea angustata* and *Crocus sativus* L. (saffron) administration, FBG levels were lower than those of the MOD group (Figure 2D and Figure S1C). Moreover, LLKL_H were found to exert the highest hypoglycaemic effect and the LLKL_M had a more promising hypoglycaemic effect than the individual herbs.

The results of OGTT performed after the 6‐week treatment showed that the blood glucose values peaked at 60 minutes after glucose administration and then decreased in all the groups (Figure 2E and Figure S1D). Figure 2G and Figure S1F depicts the glucose levels during ITT. Blood glucose values decreased in all groups after insulin injection. The OGTT and ITT curves showed that glucose and insulin tolerances were significantly improved after LLKL_H, LLKL_M, LLKL_L, MET, *Edgeworthia gardneri* (Wall.) Meisn., *Sibiraea angustata* and *Crocus sativus* L. (saffron) supplementation, respectively, compared with that of the MOD group. LLKL_H presented a most effective group, whereas LLKL_M showed a better effect than the individual herbs. Moreover, OGTT‐_AUC_ and ITT‐_AUC_ were dramatically increased in the MOD group, as compared with the NC group, whereas all the treatment groups exhibited a substantial reduction (Figure 2F,H and Figure S1E,G). Notably, the LLKL_H group showed a lower OGTT‐_AUC_ than the LLKL_M, LLKL_L and MET groups (Figure 2F) and a lower ITT‐_AUC_ than the LLKL_L and MET groups (Figure 2H). The LLKL_M group presented lower OGTT‐_AUC_ and ITT‐_AUC_ values than those of the *Edgeworthia gardneri* (Wall.) Meisn., *Sibiraea angustata* and *Crocus sativus* L. (saffron) groups (Figure S1E,G).

Serum insulin level markedly increased in the MOD group compared with those in the NC group, and this result could be reversed by both LLKL and MET treatments (Figure 1I). Additionally, we calculated HOMA‐IR index in each group and the results showed that HOMA‐IR from the MOD group was significantly increased compared with that of the NC group, whereas MET administration decreased HOMA‐IR and LLKL decreased HOMA‐IR in a dose‐dependent manner (Figure 1J). *Edgeworthia gardneri* (Wall.) Meisn., *Sibiraea angustata* and *Crocus sativus* L. (saffron) groups also exhibited a decreased in serum insulin level and HOMA‐IR, but higher than those of the LLKL_M group (Figure S1H,I). Taken together, these data provide strong evidence to suggest that the LLKL treatment improves the glycaemic control and insulin resistance in ZDF rats. Importantly, the combine use of *Edgeworthia gardneri* (Wall.) Meisn., *Sibiraea angustata* and *Crocus sativus* L. (saffron) elicits a more beneficial effect than the administration of the individual herbs.

### 3.3 Overall structural changes of the gut microbiota in response to LLKL treatment

To investigate the effects of LLKL on the gut microbiome, we performed sequencing of the V3‐V4 region of 16S rDNA sequences present in the faeces collected at the end of week 6 from the NC, MOD, LLKL_H and LLKL_M treatment groups using Illumina MiSeq. After removing low‐quality sequences, a total of 903,965 high‐quality sequences and 379 OTUs were obtained from 24 samples at a 97% homology cut‐off for subsequent analysis. The rarefaction curves indicated that although new rare phylotypes arose with additional sequencing, most of the diversity was already captured (Figure S2A,B).

**Figure 3:**
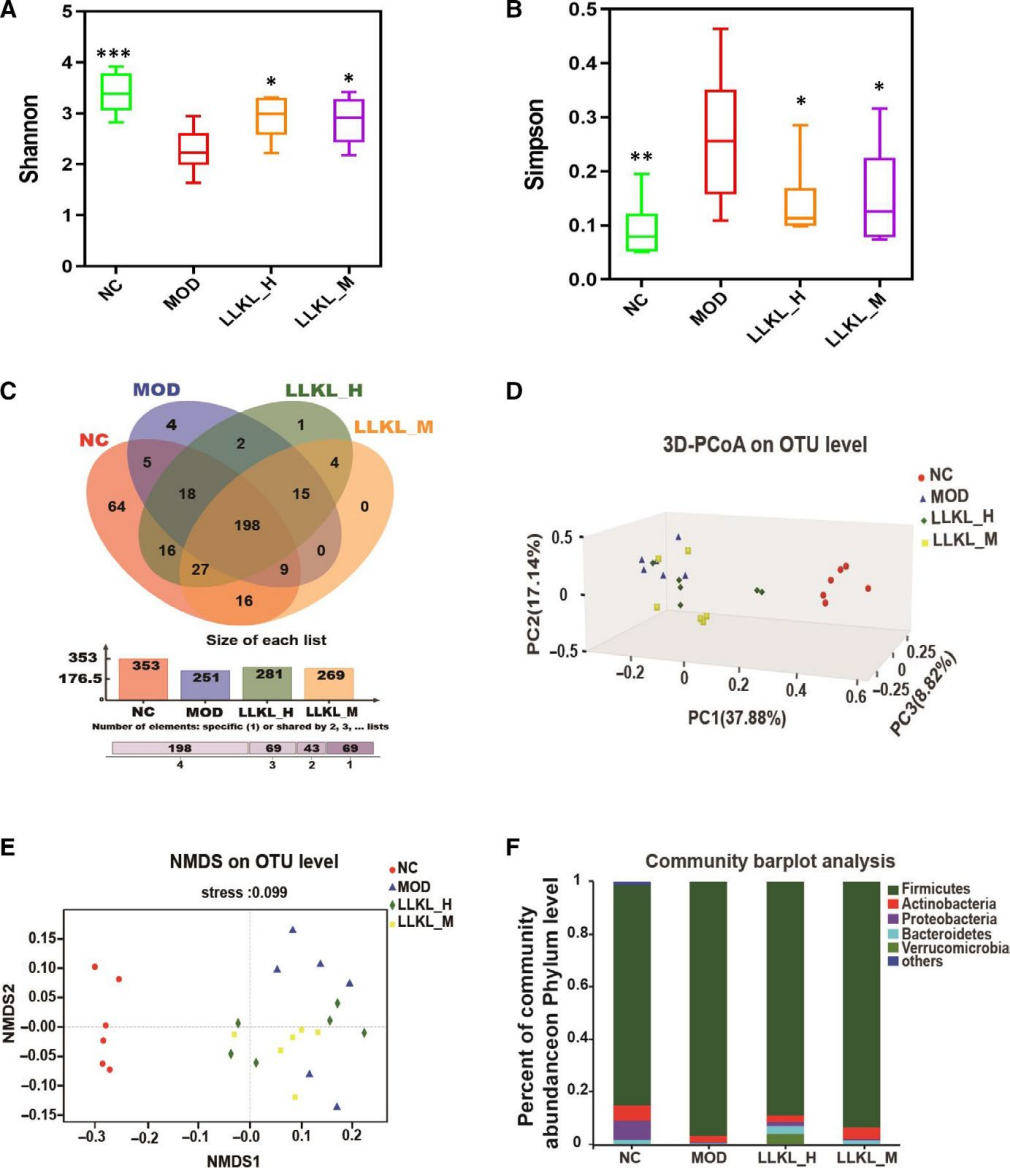
Effect of LLKL treatment on gut microbiome composition of ZDF rats. A, Shannon index in four groups. B, Simpson index in four groups. C, Venn diagrams showing the OTUs in NC, MOD, LLKL_H and LLKL_M groups. D, PCoA of an unweighted UniFrac distance matrix of the four groups at the OTU level. E, NMDS of a Bray–Curtis distance matrix of the four groups at the OTU level. F, Taxonomic composition of gut microbiome in the rats at the phylum level. Data expressed as mean ± SD (n = 6, *P < .05, **P < .01, ***P < .001 vs the MOD group)

The α‐diversity analysis of the intestinal contents showed that the Shannon value in the MOD group was significantly lower and Simpson value was markedly higher than those in the NC group, indicating that the gut microbiome diversity of the MOD group rats was lower than that in the NC group (Figure 3A,B). As expected, compared with the MOD group, the Shannon and Simpson values increased and decreased, respectively, in both LLKL_H and LLKL_M groups (Figure 3A,B), revealing that the α‐diversity increased after LLKL administration. There were 198 shared OTUs for 4 groups, and 425, 301, 346 and 330 OTUs for the NC, MOD, LLKL_H and LLKL_M groups, respectively, were obtained (Figure 3C).

To analyse the β‐diversity of the gut microbiome, principal co‐ordinates analysis (PCoA) and non‐metric multi‐dimensional scaling analysis (NMDS) based on the OTU profiles were conducted. As shown in Figure 3D,E, the gut microbiota structure of the LLKL_H and LLKL_M groups shared the same tendency and both were closer to that of the NC group compared with the MOD group, suggesting that the gut microbiota structure of LLKL_H and LLKL_M groups recovered to that of the NC group.

Next, we investigated the bacterial composition of each different group at taxonomic level. At the phylum level, as shown in Figure 3F, the top 5 phyla in the 4 groups were *Firmicutes*, *Actinobacteria*, *Proteobacteria*, *Bacteroidetes* and *Verrucomicrobia*. Compared with the NC group, the gut microbiome in the MOD group was shifted with a reduced abundance of *Bacteroidetes* and an increased proportion of *Firmicutes*, respectively, thus increasing the *Firmicutes*/*Bacteroidetes* ratio, whereas LLKL_H and LLKL_M treatment reduced this difference. In addition, compared with the NC group, the content of *Proteobacteria* and *Actinobacteria* was decreased in the MOD group. In contrast to the MOD group, the LLKL treatment groups showed increased numbers of *Proteobacteria* and *Actinobacteria*. These findings indicate a beneficial role of LLKL in maintaining gut microbiota homeostasis that results in a increased content of intestinal Gram‐negative bacteria.

### 3.4 LLKL administration diminishes the intestinal epithelial barrier damage, decreasing the levels of LPS and inflammatory cytokines

**Figure 4:**
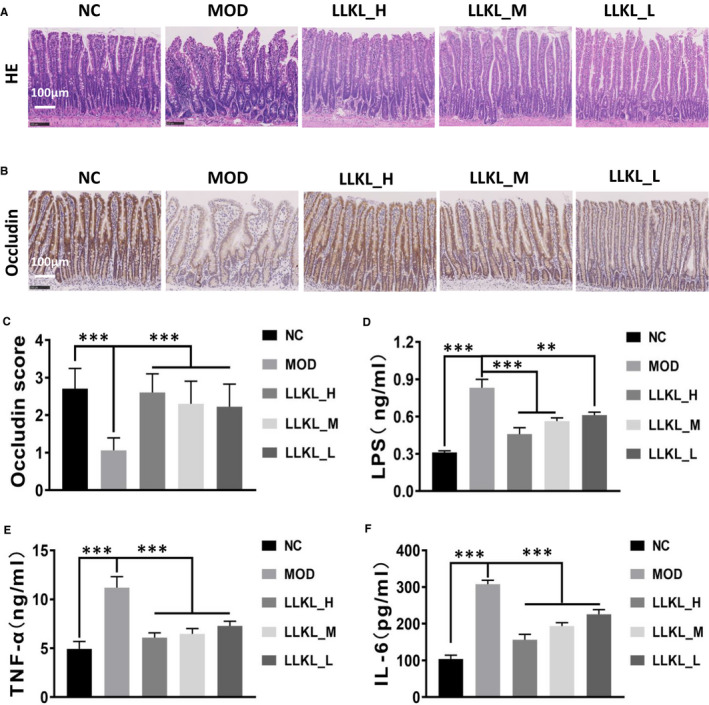
Effect of LLKL on Intestinal tissue, LPS and inflammatory cytokines. A, Intestinal tissue specimens from different treatment groups were subjected to H&amp;E staining. Representative images were presented. Scale bar represents 100 μm. B, Representative immunohistochemistry images of tissues stained with occludin antibody in the NC, MOD, LLKL_H, LLKL_M and LLKL_L groups. Scale bar represents 100 μm. C, Occludin expression score was evaluated in NC, MOD, LLKL_H, LLKL_M and LLKL_L groups (n = 4‐6). Serum levels of LPS (D), TNF‐α (E) and IL‐6 (F) (n = 6). All data were shown as means ± SD (**P < .01, ***P < .001 vs the MOD group)

LPS is produced during the constant breakdown of intestinal Gram‐negative bacteria and can translocate from the intestine to several organs, such as the liver and brain, which could further induce an inflammatory reaction. Thus, this evidence prompts us to investigate whether LLKL has a beneficial effect role on LPS in improving insulin resistance. First, we conducted HE staining to examine the effects of LLKL on small intestine and found that the MOD group displayed abnormal morphological alterations, characterized by the loss of normal villus structure of the small intestine epithelium, including disorganized, collapsed and lower villus height and crypt depth.However, these disorders were restored after LLKL treatment (Figure 4A), suggesting that LLKL diminishes the intestinal epithelial villus damage in the intestinal epithelium. Subsequently, to test the possibility that LLKL affected the small intestine epithelial permeability, we performed IHC staining to measure the expression levels of tight junction protein. As shown in Figure 4B,C, the occludin expression was diminished in the MOD group, compared with that in the NC group, but increased after LLKL administration. Interestingly, a comparison of the serum LPS and inflammatory cytokine levels showed elevated LPS, IL‐6 and TNF‐α levels in the MOD group, whereas LLKL treatment significantly reduced LPS, IL‐6 and TNF‐α levels in a dose‐dependent manner (Figure 4D‐F). These results support the idea that intestinal epithelial barrier damage could be diminished by administration of LLKL to ZDF rats, resulting in a reduced LPS and inflammatory cytokines release from the gut into the bloodstream.

### 3.5 ***LLKL treatment alters the hepatic transcriptome and inhibits the Toll*** ‐***like receptor signalling pathway***

**Figure 5:**
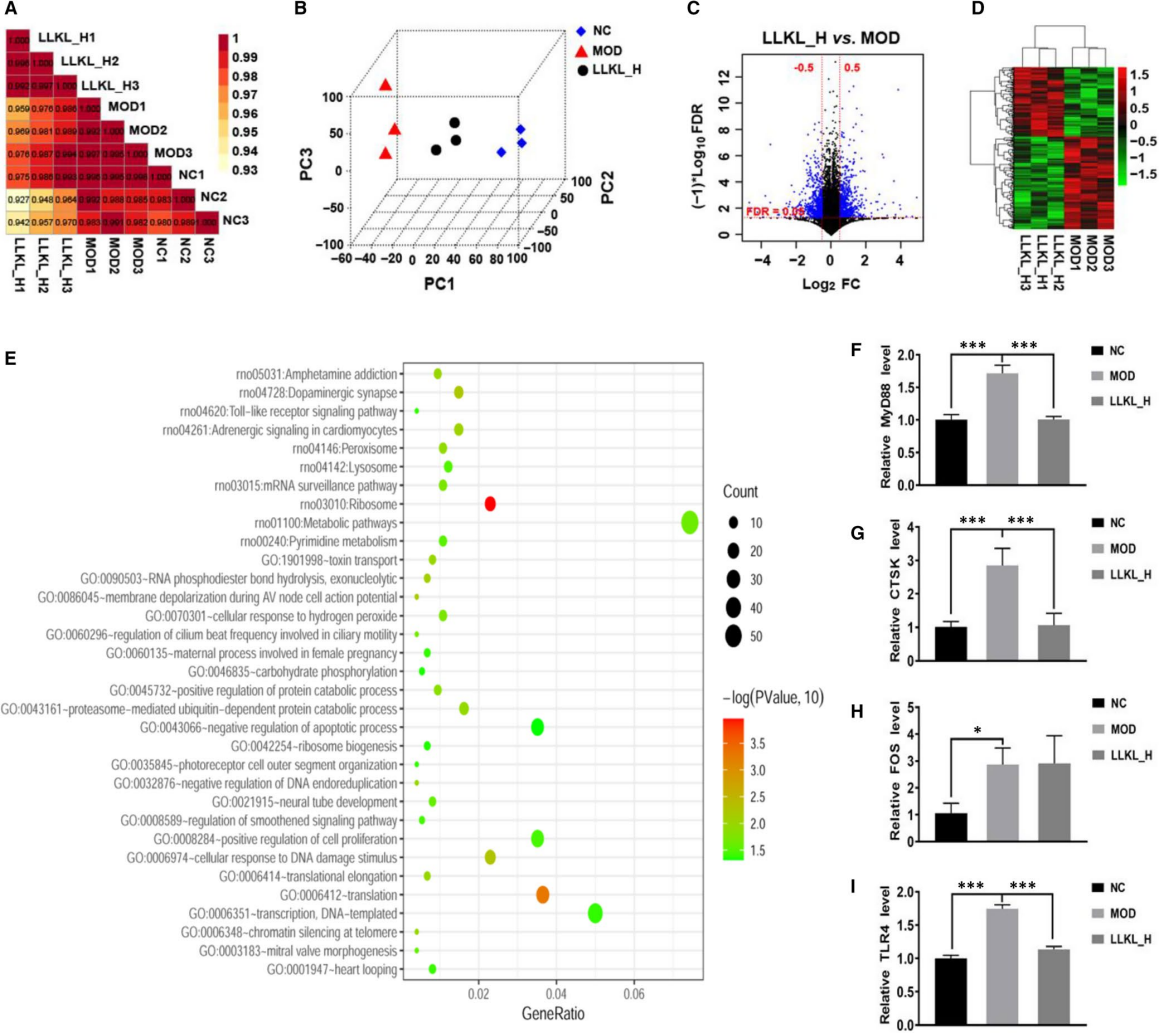
Transcriptomic profiling of the liver. A, PCoA between the NC, MOD and LLKL_H groups. B, PCA between the NC, MOD and LLKL_H groups. C, Volcano plot of the DEG between the LLKL_H and MOD groups. Blue colour points indicated the DEG, the red dashed horizontal line indicated the FDR < 0.05, and the red perpendicular dotted line indicated the |log_2_FC| > 0.5. D, A cluster heatmap of expression DEGs profiles between LLKL_H and MOD groups. E, Analysis of the GO and KEGG pathways by DAVID online tool. Analysis of MyD88 (F), FOS (G), CTSK (H) and TLR4 (I) mRNA expression in NC, MOD and LLKL_H groups by RT‐qPCR. All data were shown as means ± SD (n = 3, *P < .05, ***P < .001 vs the MOD group)

The liver is one of the most important organs for glucose and lipid metabolisms. Therefore, to further explore the hypoglycaemic mechanism of LLKL, a liver transcriptome analysis in the NC, MOD and LLKL_H groups was performed. We calculated Pearson's correlation coefficient to evaluate the association between samples and the result showed that the samples clustered closely with the treatments (Figure 5A). Consistently, the PCA demonstrated a clear separation of the NC, MOD and LLKL_H groups (Figure 5B), suggesting the high quality of our transcriptome data. Next, we identified 1495 (FDR < 0.05 and |logFC|> 0.5) and 740 DEGs, when comparing the MOD vs NC (Table S1) and the LLKL_H vs MOD groups (Table S2), respectively. The volcano plot and cluster heatmap of the differentially expressed mRNAs are shown in Figure S3A,B and Figure 5C,D. To further analyse the functional importance of the DEGs that responded to the LLKL_H treatment, we performed a functional annotation analysis by using DAVID online tool. Our results indicated that the Toll‐like receptor signalling pathway, a key pathway between the gut microbiota and the host, was significantly enriched among the top 10 KEGG pathways (Figure 5E). Specifically, there were three significantly altered genes in Toll‐like receptor signalling pathway, of which MyD88 and CTSK showed an apparently decreased expression in the LLKL_H group as compared to the MOD group, whereas FOS was up‐regulated by LLKL_H treatment. Further verification by RT‐qPCR showed that the expression of MyD88 and CTSK was increased in MOD group as compared with the NC group, but was decreased by LLKL_H treatment as compared with the MOD group (Figure 5F,G). On the contrary, FOS was up‐regulated in the MOD group as compared with the NC group, but there was no significant difference between the MOD group and the LLKL_H treatment group (Figure 5H). As previous studies have demonstrated that TLR4 responses to LPS, we also measured the expression of TLR4 even though our transcriptome data showed that compared with the MOD group, TLR4 exhibited a relative reduction but not significantly in the LLKL_H group. As shown in Figure 5I, the expression of TLR4 was significantly increased in the MOD group and decreased in the LLKL_H treatment group. These data provide evidence to suggest that the Toll‐like receptor signalling pathway plays an essential role in regulating the gut‐liver axis activation upon LLKL treatment.

**Figure 6:**
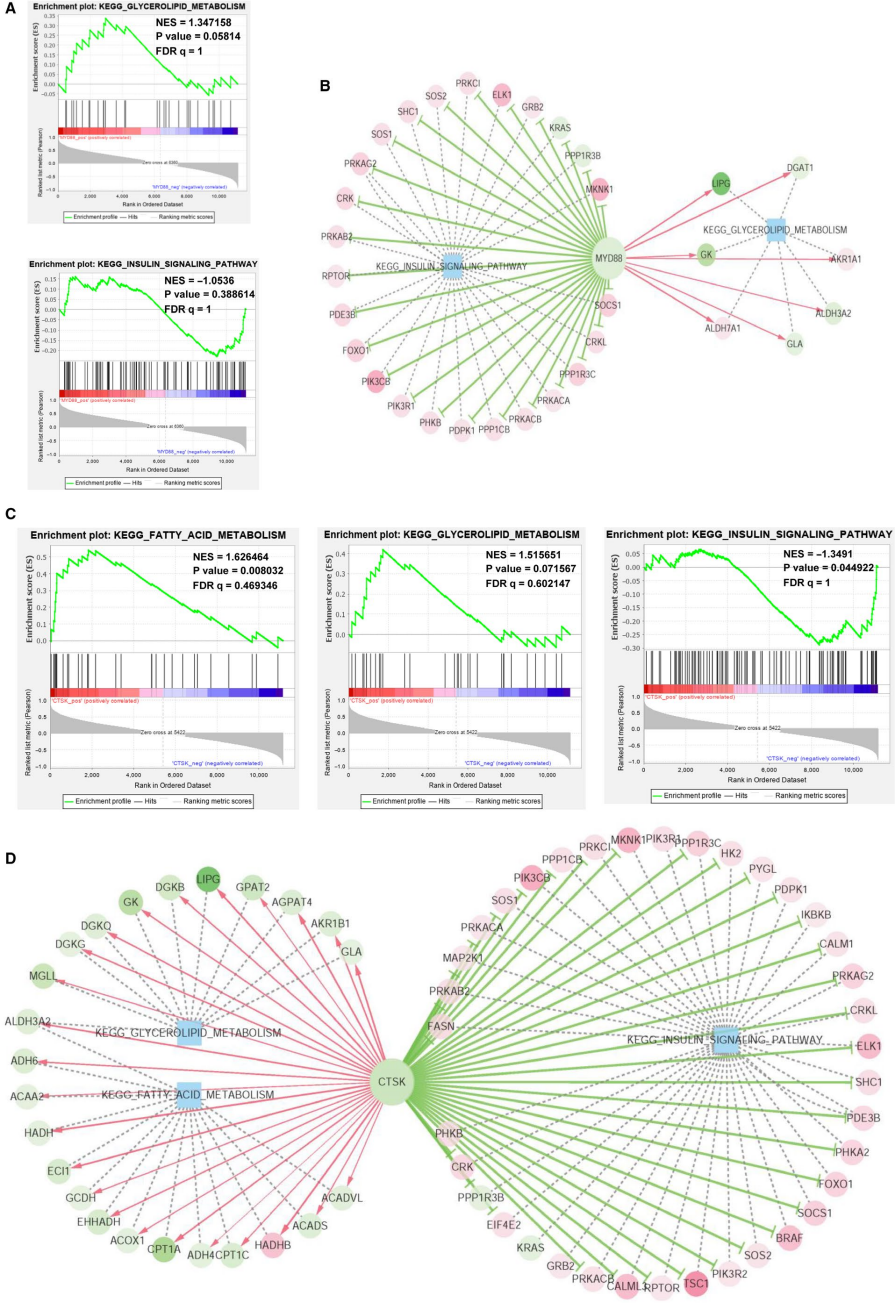
GSEA analysis and gene‐pathway regulatory network in the liver. A, MyD88 positively correlated with glycerolipid metabolism and negatively correlated with insulin signalling pathway. B, Regulatory Network visualization between MyD88 regulated gene sets and the key regulated signalling pathways. C, CTSK positively correlated with glycerolipid metabolism and fatty acid metabolism and negatively correlated with insulin signalling pathway. D, Regulatory Network visualization between CTSK regulated gene sets and the key regulated signalling pathways

In addition, GSEA was performed to future explore the potential pathways by which MyD88 and CTSK regulate the hepatic metabolism in respond to LLKL treatment based on liver expression profile data of the NC, MOD and LLKL_H groups. Interestingly, as shown in Figure 6A,C, the high MyD88 expression was positively correlated with the glycerolipid metabolism (NES = 1.347158, *P* = .05814, FDR q = 1) and negatively correlated with the insulin signalling pathway (NES = −1.0536, *P* = .388614, FDR q = 1), whereas the high CTSK expression was positively correlated with the fatty acid metabolism (ES = 1.626464, *P* = .008032, FDR q = 0.469346) and the glycerolipid metabolism (ES = 1.515651, *P* = .071567, FDR q = 0.602147) and negative correlated with the insulin signalling pathway (ES = −1.3491, *P* = .044922, FDR q = 1). These results suggested that the insulin signalling pathway was enriched and up‐regulated in the LLKL_H treatment group, whereas the glycerolipid metabolism and fatty acid metabolism were implicated and down‐regulated in the LLKL_H treatment group. Moreover, a series of regulated genes in those signalling pathways were identified and the gene‐pathway regulatory networks were constructed based on the GSEA analysis (Figure 6B,D and Table S3). Thus, these data evidently demonstrate that LLKL treatment promotes the insulin signalling pathway and inhibits glycerolipid metabolism and fatty acid metabolism.

### 3.6 LLKL ameliorates the histological, glucose and lipid metabolic features in ZDF rat livers

**Figure 7:**
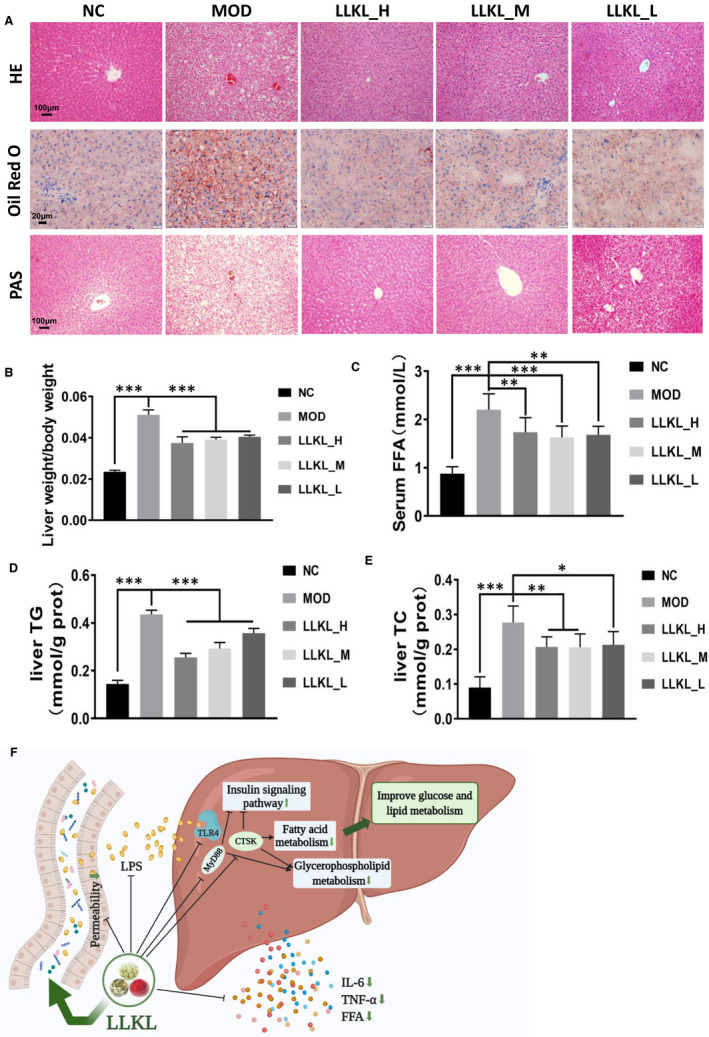
Effects of LLKL on hepatic glucose and lipid metabolism. A, Histopathological examination, Oil Red O and PAS staining of liver tissues. Representative images were presented. Scale bar represents 100 or 20 μm. B, Liver weight/BW. C, Serum levels of FFA. Levels of liver TC (D) and liver TG (E). F, Model depicts the improvement of diabetic features in ZDF rats after LLKL intake. This figure was created with BioRender.com. All data are presented as means ± SD (n = 6, *P < .05, **P < .01, ***P < .001 vs the MOD group)

To verify the histological, glucose and lipid metabolism characters in liver by biochemical experiments, the related parameters were analysed in livers of rats from the different treatment groups (Figure 7A). Compared with the NC group, HE and Oil Red O stainings revealed an obvious increase in the number and size of the lipid droplets, and morphology injury in the MOD group which were reversed by the LLKL treatment. In addition, PAS staining of the liver sections showed significantly lower glycogen deposition in the MOD group than in the NC group, whereas administration of LLKL promoted glycogen accumulation. Consistently, the liver weight index was enhanced in the MOD group and decreased in the LLKL treatment (Figure 7B). Moreover, compared with the NC group, the levels of serum FFA, liver TC and liver TG in the MOD group were notably elevated, and these effects were reversed by LLKL treatment (Figure 7C‐E). Therefore, these results reveal an effectively protective role of LLKL on hepatic morphology recovery and lipid metabolism in ZDF rats. Our data collectively led us to conclude that LLKL regulates gut microbiota and gut‐liver axis which might contribute to the anti‐diabetic effect of LLKL (Figure 7F).

## 4 DISCUSSION

T2DM has grabbed increasing research attention worldwide to develop more effective therapeutic medicine, especially those from natural products.^35^ In the present study, our results demonstrated that LLKL treatment improved insulin resistance in ZDF rats (Figure 2) and the LLKL treatment showed a better effect than the individual herbs (Figure S1). By performing a 16S rDNA sequencing analysis, we noted a disordered gut microbiota in diabetic rats, which was sufficiently improved after administration of LLKL, allowing the maintenance of homeostasis of the gut (Figure 3). Also, LLKL treatment inhibited serum LPS and inflammation, improving the intestinal structure and function (Figure 4). Hepatic transcriptome results revealed that Toll‐like receptor signalling pathway was involved in the response to LLKL treatment. RT‐qPCR results validated that LLKL decreased the expression of TLR4, MyD88 and CTSK in liver tissue (Figure 5). GSEA analysis showed an obvious enrichment of the fatty acid metabolism, glycerolipid metabolism and insulin signalling pathway (Figure 6), indicating a significant metabolism alter in liver upon LLKL treatment. Notably, our biochemical results confirmed that LLKL intake reduced liver injury and lipid accumulation, whereas it increased glycogen and decreased serum FFA, liver TC and liver TG levels (Figure 7).

TCM has been used for the treatment of T2DM for many years. LLKL, a TCM formula, was designed by Professor Tonghua Liu in Beijing University of Traditional Chinese Medicine to ameliorate T2DM in a patient population at a dose of 26 g/kg/d based on his rich clinical experiences with the guidance of Chinese medicine theory. This is the first study that evaluated the anti‐diabetic efficacy and underlying mechanism of LLKL in ZDF rats. We found that administration of LLKL, *Edgeworthia gardneri* (Wall.) Meisn., *Sibiraea angustata* and *Crocus sativus* L. (saffron), effectively decreased FBG, OGTT‐_AUC_, ITT‐_AUC_ and FINS levels and improved insulin resistance. Our results were consistent with those of previous studies. For example, Ma et al^36^ demonstrated that effective components of *Edgeworthia gardneri* (Wall.) Meisn. significantly reduced FBG level in STZ‐induced diabetic mice. Xiao et al^37^ reported that *Sibiraea angustata* may be used as a safe and effective nutraceutical to manage obesity. Milajerdi, A. proved that *Crocus sativus* L. (saffron) hydroalcoholic extract treatment decreased FBG level in T2DM patients.^38^ Moreover, the LLKL_M group showed the lowest FBG, OGTT‐_AUC_, ITT‐_AUC_, FINS and HOMA‐IR values among LLKL_M, *Edgeworthia gardneri* (Wall.) Meisn., *Sibiraea angustata* and *Crocus sativus* L. (saffron) groups, providing evidence to support the assumption that the LLKL formula have more promising beneficial anti‐diabetic effects than the individual herbs. In addition, the levels of OGTT‐_AUC_ and ITT‐_AUC_ in the LLKL_H group were lower than those in the MET group, indicating that the high dose of LLKL had a better hypoglycaemic effect, which might better than 0.135 g/kg/d metformin. Importantly, TCM is a natural medicine that is widely used due to its minimum side‐effects, effectiveness and low toxicity, compared with other anti‐diabetic drugs. Furthermore, the doses of the three herbs that constituted our LLKL formula were lower than those reported in other studies. For example, to study the therapeutic effect of *Edgeworthia gardneri* (Wall.) Meisn. on hyperglycaemia animal model, three doses of *Edgeworthia gardneri* (Wall.) Meisn. (50, 25 and 12.5 g/kg) were used to treat hyperglycaemia mice.^39^ To investigate the effect and mechanism of *Sibiraea angustata* on lipid metabolism, 1.05, 2.1 and 4.2 g/kg *Sibiraea angustata* were intragastrically administered to obese SD rats for 8 weeks.^40^ Also, oral administration of *Crocus sativus* L. ethanolic extracts at doses up to 5 g/kg did not cause any mortalities or signs of toxicity in mice.^41^ Taken together, our data indicate that LLKL effectively decreases the glycaemic levels and improves insulin resistance in ZDF rats.

Accumulating studies have revealed a correlation between diabetes and altered gut microbiota composition and diversity, suggesting a pivotal role of gut microbiota in the development of an anti‐diabetes treatment strategy.^42^ For example, probiotics have been identified as effective adjuvants to improve insulin resistance due to their gut microbiota regulation function.^43^ Recent studies reported an increased ratio of *Firmicutes* to *Bacteroidetes* in both T2DM animal model and patients, which was positively correlated with FBG level and the inflammation status.^44^, ^45^, ^46^ Although the gut microbiota plays an important role in diabetes, the effect of LLKL on gut microbiota in ZDF rats is still unknown. In this study, we demonstrated that the bacterial diversity which decreased in ZDF rats was restored by LLKL treatment. In addition, we noted that *Firmicutes* and *Bacteroidetes* were the two most abundant populations in ZDF rats, which was consistent with previous studies.^44^, ^45^ Importantly, compared with the NC group, the gut microbiome in the MOD group showed a reduced abundance of *Bacteroidetes* and an increased proportion of *Firmicutes*, which resulted in an increase *Firmicutes*/*Bacteroidetes* ratio, whereas LLKL_H and LLKL_M treatments sufficiently reduced these differences. This result is consistent with those obtained by Zhang et al^47^ in which the treatment of diabetic mice with *Edgeworthia gardneri* (Wall.) Meisn. water extract significantly enriched *Bacteroidetes* and suppressed *Firmicutes*. In addition, our data showed that the number of *Proteobacteria* in the MOD group decreased and increased in the LLKL treatment group, and in contrast to the results obtained by Zhang et al study, but consistent with other reports.^48^, ^49^ This evidence suggests that LLKL promotes the balance of gut microbiota in ZDF rats.

Most of the species belonging to the *Firmicutes* phylum are Gram‐positive and most species belonging to the Bacteroidetes phylum are Gram‐negative.^50^ Recent studies have revealed that the level of LPS, a component present in the outer membrane of Gram‐negative bacteria,^51^ was higher in presence of diabetes due to a decreased of *Bacteroidetes* to *Firmicutes* ratio, which further induced the release of pro‐inflammatory cytokines in blood and tissues, leading to insulin resistance and diabetes, particularly by reducing hepatic insulin action.^52^, ^53^ In addition, it was reported that red pitaya betacyanins prevented the elevation of FBG and serum inflammatory factors’ levels with the decreased ratio of *Firmicutes* to *Bacteroidetes* in diabetic mice.^54^ Moreover, altered intestinal microbiota contributed to the decrease in gut tight junction proteins, which further increased the intestinal permeability, leading to excessive translocation of LPS and inflammatory cytokines into the circulation system.^55^, ^56^ In the present study, diabetic rats displayed abnormal morphological alterations, characterized by the loss of the normal villus structure in the small intestine epithelium, including disorganized, collapsed villi and decreased expression of occludin, which are consistent with previous studies.^57^ As expected, LLKL treatment significantly reversed these changes. Accordingly, we also found that LLKL treatment alleviated the serum levels of LPS, IL‐6 and TNF‐α compared with those of the diabetic rats. These results indicate that the balance of the gut microbiota and the anti‐inflammatory activity may be involved in the mechanism by which LLKL ameliorates diabetes in ZDF rats.

The Toll‐like receptor signalling pathway plays an important role in the gut and liver crosstalk in metabolic disorders, such as NAFLD, diabetes and obesity.^58^, ^59^, ^60^ TLR4 responds to ligands such as LPS, also referred to as endotoxin, and fatty acids, and initiates a response by forming a complex with myeloid differentiation factor 2 (MD‐2), which leads to activation of both MyD88‐dependent and non‐MyD88‐dependent signalling cascades.^61^ In addition, MyD88 is considered to be a central hub of the inflammatory signalling cascades,^62^ by inducing the transcription of pro‐inflammatory cytokines,^18^ mediating energy, lipid and glucose metabolism.^62^, ^63^ The accumulation of glycerolipid metabolites has been implicated in the pathogenesis of hepatic insulin resistance. In our study, a profile of the hepatic transcriptome indicated an enrichment of the Toll‐like receptor signalling pathway. TLR4, MyD88 and CTSK expressions were restrained by LLKL administration, which contributed to the down‐regulation of fatty acid metabolism and glycerolipid metabolism and up‐regulation of the insulin signalling pathway. Moreover, according to our results, hepatic morphology, excessive hepatic lipid deposition and decreased glycogen were alleviated by LLKL supplementation. In addition, LLKL effectively inhibited serum FFA, liver TC and liver TG levels. Thus, LLKL may alleviate insulin resistance and regulate liver metabolism via the gut‐liver crosstalk.

There are a few limitations to our experiments. Although we determined that LLKL inhibited MyD88 and CTSK expression to down‐regulate the fatty acid metabolism and glycerolipid metabolism and up‐regulated insulin signalling pathway through a series of genes, further efforts are necessary to study its precise molecular mechanisms. Other factors, including increased insulin sensitivity in muscle and adipocytes and protection of pancreatic β‐cells, might also be involved in the underlying mechanism of anti‐diabetes in LLKL. Further experiments are needed to comprehensively explore the underlying mechanisms of LLKL. In addition, to further verify the effect of LLKL on gut microbiota, we will perform faecal microbiota transplantation experiments in our future study. Additionally, we will further study the effect and metabolic processes of LLKL on the gut microbiota of T2DM patients. Overall, our data contribute to the understanding of the anti‐diabetic effects and the underlying mechanisms of LLKL in ZDF rats.

In summary, our study indicates that LLKL elicited anti‐diabetic properties in ZDF rats. In particular, its mechanism of action is mediated by modulating the gut microbiota dysbiosis and gut‐liver axis activation via the Toll‐like receptor signalling pathway. Taken together, these findings provide new evidence and insights into the anti‐diabetic effects of LLKL.

## CONFLICT OF INTEREST

The authors declare that they have no conflict of interest.

## AUTHOR CONTRIBUTIONS

**Mei Li:** Conceptualization (equal); Data curation (equal); Formal analysis (equal); Investigation (equal); Methodology (equal); Writing‐original draft (lead); Writing‐review & editing (equal). **Lei Ding:** Data curation (supporting); Investigation (supporting); Writing‐original draft (supporting); Writing‐review & editing (supporting). **Yu‐Li Hu:** Data curation (supporting); Investigation (supporting); Writing‐original draft (supporting); Writing‐review & editing (supporting). **Ling‐Ling Qin:** Investigation (supporting). **You Wu:** Investigation (supporting). **Wei Liu:** Investigation (supporting). **Li‐Li Wu:** Investigation (supporting); Methodology (supporting); Project administration (equal); Supervision (equal); Writing‐original draft (supporting); Writing‐review & editing (supporting). **Tong‐Hua Liu:** Conceptualization (lead); Funding acquisition (lead); Methodology (lead); Project administration (lead); Resources (lead); Supervision (equal); Writing‐original draft (supporting); Writing‐review & editing (supporting).

## Supporting information

Fig S1

Fig S2

Fig S3

Table S1

Table S2

Table S3

Supplementary Material

## Data Availability

The data that support the findings of this study are available from the corresponding author upon reasonable request.
