# Supplementary figures and images for: Herbal formula LLKL ameliorates hyperglycaemia, modulates the gut microbiota and regulates the gut‐liver axis in Zucker diabetic fatty rats
Source: J Cell Mol Med. 2020 Nov 20;25(1):367–82. doi: 10.1111/jcmm.16084 (PMC7810939; doi:10.1111/jcmm.16084)

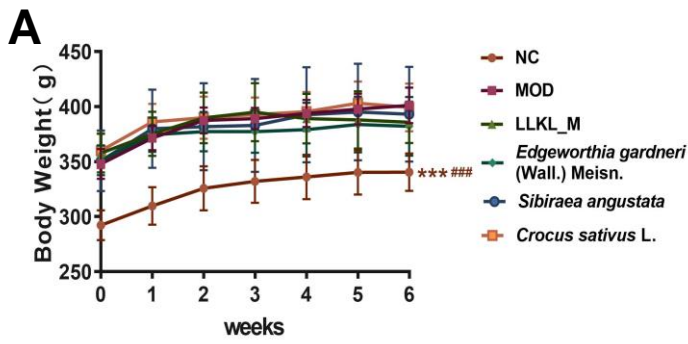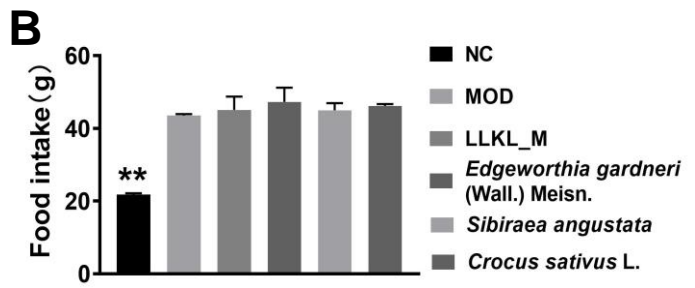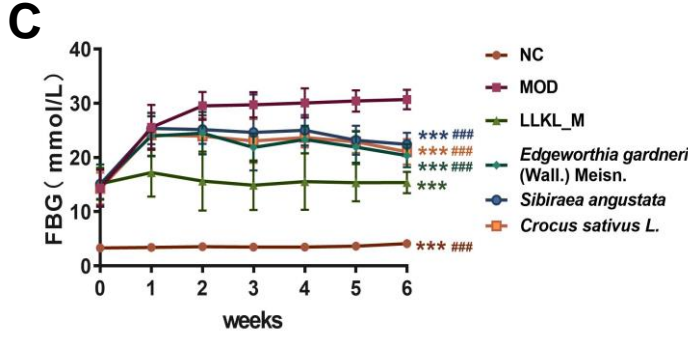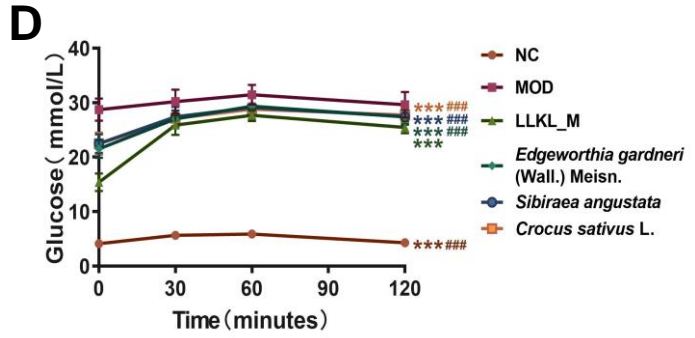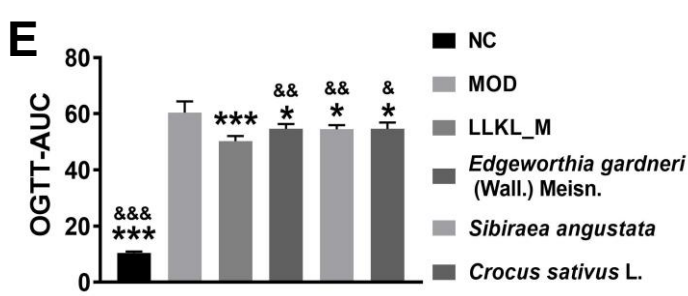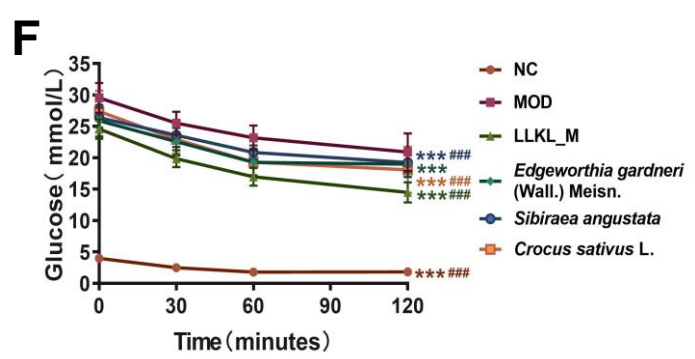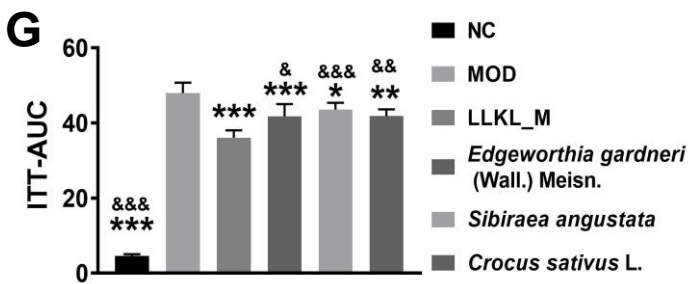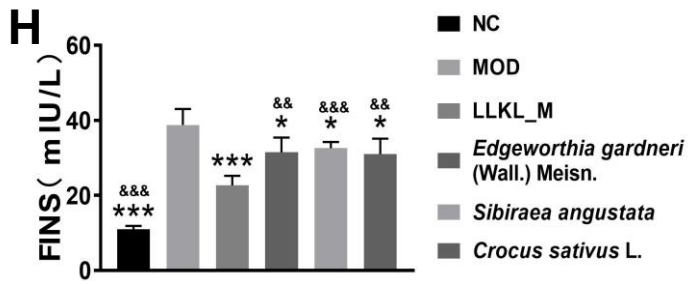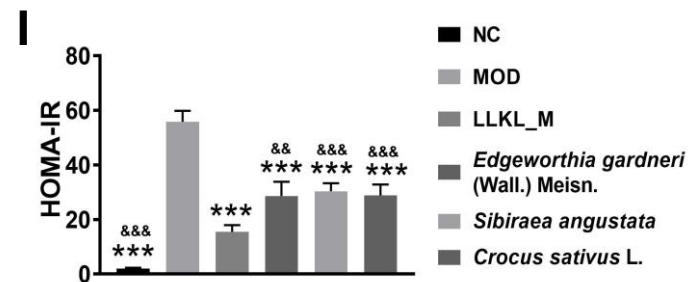

Supplement: Supplementary file 1 — Fig S1 [file JCMM-25-367-s001.pdf]

**A**

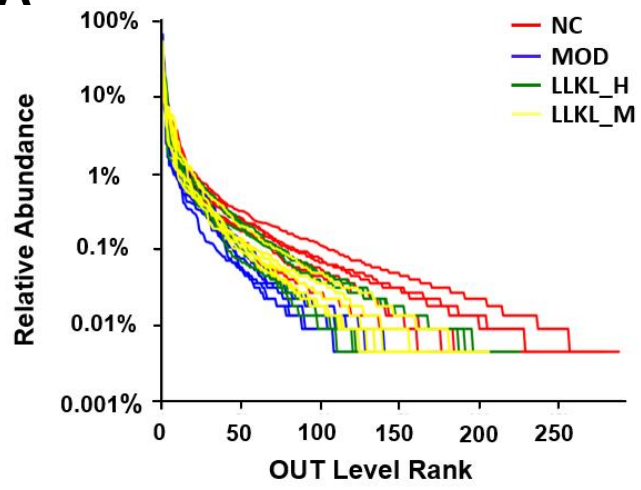

**B**

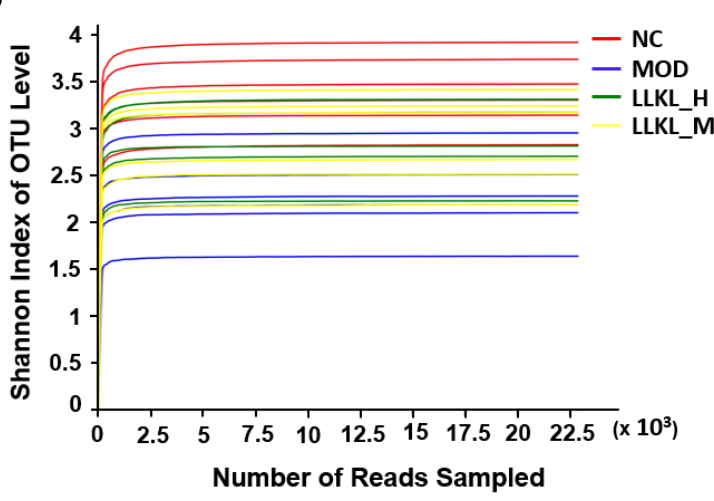

Supplement: Supplementary file 2 — Fig S2 [file JCMM-25-367-s002.pdf]

A

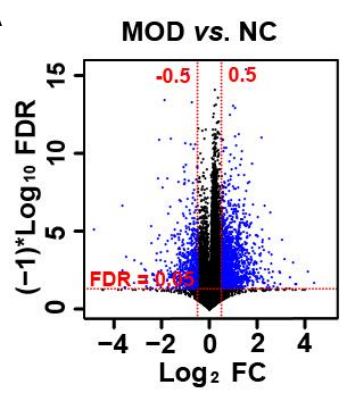

B

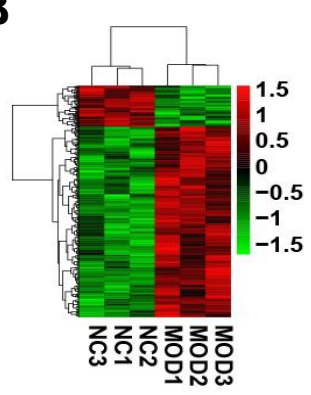

Supplement: Supplementary file 3 — Fig S3 [file JCMM-25-367-s003.pdf]
