# Supplementary material for: Herbal formula LLKL ameliorates hyperglycaemia, modulates the gut microbiota and regulates the gut‐liver axis in Zucker diabetic fatty rats
Source: J Cell Mol Med. 2020 Nov 20;25(1):367–82. doi: 10.1111/jcmm.16084 (PMC7810939; doi:10.1111/jcmm.16084)
Supplement: Supplementary file 7 — Supplementary Material [file JCMM-25-367-s007.docx]

**Supplemental Figure Legends**

**Supplemental *Figure 1. Edgeworthia gardneri (Wall.) Meisn.,*** ***Sibiraea angustata and*** ***Crocus sativus L. treatment alleviates insulin resistance in ZDF rats.***

(A) BW, (B) Food intake, (C) FBG, (D) Curve of OGTT, (E) AUC of OGTT, (F) Curve of ITT, (G) AUC of ITT, (H) FINS and (I) HOMA-IR of NC, MOD, LLKL_M, *Edgeworthia gardneri* (Wall.) Meisn.*, Sibiraea angustata and Crocus sativus* L*.* treatment groups. Data were shown as means ± SD (n = 8, **P* < 0.05, ***P* < 0.01, ****P* < 0.001 *versus* the MOD group; & *P* < 0.05, && *P* < 0.01, &&& *P* < 0.001 *versus* the LLKL_M group).

**Supplemental *Figure 2. Rarefaction analysis curve based on the α-diversity***

(A) Rank abundance curve at the OUT level. (B) Shannon curve analysis of the different groups at the OUT level.

**Supplemental *Figure 3.*** ***Volcano‑Plot and heatmap between MOD and NC groups***

(A) Volcano‑Plot of the DEGs between MOD and NC groups. Blue color points indicated the DEG, the red dashed horizontal line indicated the FDR < 0.05 and the red perpendicular dotted line indicated the |log2FC| > 0.05. (B) A cluster heatmap of expression profiles of DEG between MOD and NC groups.
